# Study on the Effect of the Vaginal Administration of Conjugated Estrogens Cream Combined with Progesterone on the Endometrium of Rats and Its Mechanism of Action

**DOI:** 10.3390/biomedicines12092074

**Published:** 2024-09-11

**Authors:** Panpan Li, Cenyu Xiao, Zhiyuan Lv, Haiyang Cui, Xiaoli Gao

**Affiliations:** 1 College of Pharmacy, Xinjiang Medical University, Urumqi 830011, China; m18119216991@163.com (P.L.); cenyu_xiao@163.com (C.X.); L17698951427@163.com (Z.L.); 19845732756@163.com (H.C.); 2 Xinjiang Key Laboratory of Active Components and Drug Release Technology of Natural Drugs, Urumqi 830011, China; 3 Engineering Research Center of Xinjiang and Central Asian Medicine Resources, Ministry of Education, Urumqi 830011, China

**Keywords:** vaginal drug delivery, compound preparation, conjugated estrogens, progesterone, endometrial hyperplasia

## Abstract

The purpose of this study was to investigate the impact of conjugated estrogen cream, in conjunction with progesterone, on the endometrium, following vaginal administration, and assess the combined dose–effect relationship with progesterone. Initially, bilateral ovaries from mature, female, Sprague Dawley rats were excised to establish a hypoestrogenic (perimenopausal) model. A conjugated estrogen–progesterone combination cream was administered vaginally for a duration of 12 days. Subsequently, this study used pathological sections, Enzyme-Linked Immunosorbent Assay (ELISA) for pharmacodynamic studies, network pharmacology to explore possible signalling pathways associated with the drug and menopausal syndrome, and partial validation using a real-time quantitative polymerase chain reaction (RT-qPCR) and immunohistochemistry (ICH). The results demonstrate that, relative to the model group, the conjugated estrogen monotherapy significantly increased the uterine weight coefficients (*p* < 0.0001) and endometrial thickness (*p* < 0.001) and upregulated the expression of Cyclin D1 and VEGF. Moreover, this treatment downregulated PTEN expression. The co-administration of progesterone reversed these effects in a dose-dependent manner. Overall, the vaginal administration of a combination of progesterone and conjugated estrogen cream demonstrated the ability to mitigate endometrial hyperplasia induced by conjugated estrogen vaginal cream monotherapy. Furthermore, the effect of progesterone exhibited a dose-dependent response.

## 1. Introduction

Genitourinary syndrome of menopause (GSM) has become a major factor impacting women’s health and quality of life, as a result of the increase in human life expectancy and the trend of global population ageing [1]. GSM encompasses a combination of symptoms and signs related to genital tract atrophy, urinary tract atrophy, and sexual dysfunction in women during the transition period between the menopause and postmenopause. These issues stem from reduced levels of estrogen and other sex hormones [2,3,4]. The term was first introduced by the North American Menopause Society (NAMS) and the International Society for the Study of Women’s Sexual Health (ISSWSH) in 2014 [5]. Research data indicate that the prevalence of GSM is 65% in women one year into the menopause, increasing to as high as 85% at six years postmenopause [6]. Symptoms associated with GSM encompass components of both genital symptoms and urinary symptoms. Common genital symptoms include vaginal dryness (55%), dyspareunia (44%), genital irritation (37%), and sexual functioning issues (59%) [7]. Urinary symptoms include urgency, pain, frequency, difficulty, and recurrent urinary tract infections [6]. According to the Chinese Guidelines for Menopausal Management and Menopausal Hormone Therapy (version 2023), if systemic menopausal hormone therapy (MHT) fails to completely alleviate GSM symptoms, the addition of local estrogen therapy is recommended. Specifically, local estrogen therapy is considered the most effective standard treatment for GSM, because it directly addresses the root cause of local estrogen deficiency [8,9]. It can improve moderate-to-severe vaginal atrophy symptoms, effectively preventing recurrent urinary tract infections, restoring vaginal cytology, increasing vaginal blood flow and fluid secretion, maintaining microbial homeostasis, lowering the pH, and enhancing the resistance of vaginal cells to infection and inflammation [9,10,11].

Conjugated estrogen (CE) cream is widely employed in the treatment of menopausal genitourinary syndromes and pelvic floor dysfunction disorders, such as atrophic vaginitis and vulvar dryness. In the 2023 edition of the Chinese Guidelines for the Management of Menopause and Menopausal Hormone Therapy, CE cream is recommended as a first-line therapeutic agent for the treatment of GSM. It has also been used extensively in conjunction with uterine support and pelvic floor surgery, in the use of a pelvic floor mesh, colposcopy, hysteroscopy, and the removal of intrauterine devices (IUDs). However, vaginal estrogen therapy exhibits minimal systemic absorption, potentially resulting in adverse effects, such as headaches, mastalgia, nausea, emesis, gastrointestinal discomfort, abdominal distension, and weight fluctuations [3]. In addition to this, studies have shown that the prolonged application of vaginally administered estrogen, such as CE cream, may lead to a slight thickening of the endometrium [12], which increases the risk of endometrial hyperplasia, a potential precursor to endometrial cancer. Clinical data confirming the endometrial safety of applying topical vaginal estrogen for more than one year is currently lacking [13]. Finding ways to effectively inhibit endometrial hyperplasia induced by long-term estrogen interventions and improving the safety of prolonged continuous use of estrogen cream is a crucial challenge in this field.

In order to mitigate the risk of endometrial thickening associated with the long-term application of CE cream, the incorporation of micronized progesterone (P4) during the vaginal administration of such cream may serve to inhibit endometrial thickening and enhance the safety profile of prolonged CE cream use. Studies have shown that a combination of oestradiol and progesterone is used to treat menopausal vasomotor symptoms (VMS) and GSM, while providing endometrial protection [14,15]. Therefore, the objective of this study was to assess the feasibility and efficacy of a CE–P4 combination vaginal cream. The specific goals were to formulate a combination cream, evaluate the impact of P4 on endometrial thickening, determine the optimal dosage of P4 in combination with CE cream, and utilize network pharmacology to predict and partially validate the potential signalling pathways associated with the combination treatment.

## 2. Materials and Methods

### 2.1. Drugs and Reagents

Progesterone (98%) was obtained from Hubei Gedian Humanwell Pharmaceutical Co., Ltd. (Ezhou, China). Crinone^®^ (8%) was procured from Fleet laboratories Limited (Watford, Hertfordshire, UK). CE API was supplied by Xinziyuan Biopharmaceutical Co., Ltd. (Yili, China). Quickey Pro Rat E2 (Estradiol) ELISA kit and Quickey Pro Rat Pg (Progesterone) ELISA kit were purchased from Elabscience (Wuhan, China). TRIZOL Reagent was obtained from Invitrogen (Carlsbad, CA, USA). Reverse Transcription Kit and PCR Kit were obtained from Takara Biomedical Technology Co., Ltd. (Beijing, China). Permeabilizing agent Triton x-100, endogenous peroxidase blocker, and Tris-EDTA antigen repair solution were procured from Beijing Solarbio Solepol Technology Co., Ltd. (Beijing, China). PTEN antibody was purchased from Beijing Bioss Biotechnology Co., Ltd. (Beijing, China). VEGFA antibody was purchased. from Proteintech group, lnc. (Wuhan, China). Cyclin D1 antibody was purchased from Aabcam (Cambridge, UK).

### 2.2. Preparation and Properties of the Creams

The study involved the preparation and evaluation of a CE–P4 compound vaginal cream, designed with reference to the prescription and process associated with the Honglilai^®^ CE cream. This compound cream was subjected to an assessment and subsequently utilized as a test drug for animal experiments upon meeting the required standards. Following the dosage specifications for CE in Honglilai^®^ CE cream (0.625 mg/g/d) and P4 in the Crinone^®^ vaginal preparation (90 mg/1.125 g/d), four compounded vaginal creams were formulated. These creams contained CE at a concentration of 0.625 mg/g and varying P4 concentrations of 40 mg/g, 80 mg/g, 160 mg/g, and 320 mg/g.

The appearance and rheological properties of the four creams were observed. Approximately 2 mL of each homemade cream was applied to the cone–plate mold of the KNX2110 rheometer(Malvery Instruments Ltd., London, UK). The dynamic viscosity of the samples was determined at a temperature of 25.0 °C, using a CP4/40 rotor, with a shear rate ranging from 0.1 to 100 s^−1^. The resulting dynamic viscosity–shear rate curves were recorded to illustrate the variation in dynamic viscosity with the applied shear stress at 25.0 °C.

### 2.3. Animals

Healthy, female, Sprague Dawley rats (body weight 180–220 g) were obtained from the Experimental Animal Center at Xinjiang Medical University (Animal Qualification Number SCXK 2023–0004). The experimental animals underwent a 5-day acclimatization period in the SPF (Specific Pathogen Free) animal laboratory. The laboratory maintained a temperature range of between 20 to 26 °C, with humidity levels between 50% and 60%. It featured excellent air circulation and adhered to high hygienic standards, ensuring a noise-free environment. During the acclimatization and experimental periods, the Sprague Dawley rats had free access to tap water and were provided with a normal diet. All the animal procedures were approved by the Ethics Committee of Xinjiang Medical University (IACUC-20220314-19), complying with the ARRIVE guidelines, and all experiments were carried out in accordance with the National Institutes of Health Guide for the Care and Use of Laboratory Animals (NIH Publications No. 8023, revised 1978).

### 2.4. Establishment of the Menopause Model

Forty-two female Sprague Dawley (SD) rats were selected for the study. They were anaesthetized and incisions were made on both sides, next to the midline of the back. The fallopian tubes were ligated, the fat was separated, and the ovaries were excised [16]. Penicillin was applied to the muscle layer, and the incision site was sterilized and sutured. An additional 6 female SD rats were chosen as the normal control group. They were numbered, anaesthetized, and underwent a similar procedure, with the exception that only part of the fat was surgically removed, without excising the ovaries. The remaining steps were consistent with those of the experimental group. Postoperatively, a one-week observation period was conducted, followed by vaginal smears to assess the modelling effects.

### 2.5. Administration

During the experiment, the Sprague Dawley (SD) rats were divided into eight groups. The rats that underwent a successful ovariectomy were randomly assigned to seven groups, each consisting of six rats (n = 6). The dosages for the groups were determined by referring to the human dose of CE (0.625 mg/g/d) in the CE cream (Honglilai^®^) and the human dose of P4 (90 mg/1.125 g/d) in the P4 vaginal preparation (Crinone^®^). The doses per animal were adjusted according to weight, based on those used in women with GSM, testing increasing doses of progesterone. The groupings and respective dosages were as follows: CE group (CE, 0.448 μg/g), Crinone^®^ group (P4, 57.6 μg/g), model group (blank cream), dose group 1 (CE–P4-1, 0.448 μg/g–28.8 μg/g), dose group 2 (CE–P4-2, 0.448 μg/g–57.6 μg/g), dose group 3 (CE–P4-3, 0.448 μg/g–115.2 μg/g), and dose group 4 (CE–P4-4, 0.448 μg/g–230.4 μg/g). Additionally, a rat with only fat removed from the vicinity of the ovary served as the sham-operated group, or the normal group (normal). All groups, except the normal group, received daily vaginal administration of the cream. The model group was given a blank cream without CE–P4 for two weeks. Vaginal smears were collected on the 12th day of administration for observation.

After 12 days of administration, the rats were weighed and anaesthetized. Blood was collected from the abdominal aorta and left at room temperature for 1 hour. Subsequently, it was centrifuged at 3000 revolutions per minute, for 20 minutes, to obtain serum, which was then stored in a refrigerator at −80 °C. Additionally, blood was collected from the fresh rat uterus. The fresh rat uterus, vagina, and other tissues were swiftly collected, stripped of adherent fat, cleaned of extra-uterine fluid, and weighed. The vagina and mammary gland were fixed in 4% paraformaldehyde. The uterus tissue was divided into two parts, with one part fixed in 4% paraformaldehyde and the other stored in a refrigerator at −80 °C.

### 2.6. Vaginal Cytology

For the vaginal smears [17], a cotton swab soaked in saline was inserted into the vagina of the rats, rotated several times to collect the vaginal cells, and then evenly coated on slides. The slides were allowed to air dry and were subsequently fixed with 4% paraformaldehyde for 30 minutes. After fixation, the smears were stained with melamine staining solution. After a 10-minute staining period, the slides were removed and the edges were rinsed gently with distilled water. After rinsing, the slides were air-dried and then observed and photographed under the LEICA DM 3000 LED microscope (Beijing Yuechangxing Technology Co., Ltd., Beijing, China) at 100x magnification. ImageJ version 9.0 software was utilized to count the leukocytes, keratinized cells, and nucleated epithelial cells in the sample collected on the 12th day. Further analysis was performed.

### 2.7. Serum Levels of Steroid Hormones

The serum estradiol and P4 levels in each group of rats were measured meticulously, following the enzyme-linked immunosorbent assay (ELISA) kit specifications. This involved using specific ELISA kits designed for the accurate quantification of estradiol and P4 levels in the serum samples obtained from the rats.

### 2.8. Hematoxylin–Eosin Staining

The fixed rat vagina, uterus, and mammary glands were embedded in paraffin, sliced into 5 μm thick sections, and subjected to hematoxylin–eosin staining. The stained sections were then examined under a light microscope to observe and analyze their morphological changes.

### 2.9. Network Pharmacology

The OMIM and DisGeNET databases were utilized to retrieve potential targets associated with menopausal syndrome. The targets for CE and P4 were obtained from the SwissDrugDesign and GeneCards databases. The identified targets were then used to construct a “drug–constituent target menopausal syndrome” interaction network, and the intersecting targets were imported into the STRING database to create a protein–protein interaction (PPI) network. The topology of the obtained PPI network was analyzed using Cytoscape 3.9.1 and the core targets of CE, P4, and menopausal syndrome were filtered based on the node degree. The gene names of the intersected targets were fed into the DAVID database for Gene Ontology (GO) functional enrichment analysis and Kyoto Encyclopedia of Genes and Genomes (KEGG) pathway enrichment analysis. The results were visualized using a microbiology platform. The GO database, which includes biological processes (BP), molecular functions (MF), and cellular components (CC), was applied to analyze the potential biomolecular mechanisms. The KEGG database was used to identify biological functions and candidate targets. For molecular docking, AutoDockTools (version 1.5.7) was employed to dock CE and P4 to their respective target proteins, obtaining their lowest binding energies. The molecular docking results were visualized and analyzed using PyMOL (version 1.7.2.1) and Discovery Studio (version 19.1.0) software.

### 2.10. The Cyclin D1, PTEN, and VEGF Gene Expression in Uterine Tissue

#### 2.10.1. Total RNA Isolation and Reverse Transcriptase (RT) Reaction

The total RNA from endometrial tissues was extracted utilizing the TRIZOL reagent. The quantity and quality of the total RNA were assessed using a NanoDrop 2000 ultra-micro, UV–visible spectrophotometer (Thermo Fisher Scientific, Waltham, MA, USA) at 260 nm. The RNA samples with a 260/280 nm ratio falling within the range of 1.8-2.0 were considered to be of high quality. Subsequently, reverse transcription was performed using a regular PCR machine (Mycycler, Bio-Rad) (Hercules, CA, USA) and the resulting cDNA was stored at −20 °C for subsequent use.

#### 2.10.2. Real-Time Quantitative Polymerase Chain Reaction (RT-qPCR)

The primers for VEGF, PTEN, Cyclin D1, and the stably expressed, endogenous GAPDH were procured from SangBiotech Bioengineering Co., Ltd. (Shanghai, SangBiotech). A reverse transcription polymerase chain reaction (RT-PCR) was conducted to assess the relative abundance of each transcript using the DNA-binding dye SYBR Green (Takara) and the QuantStudio 1 plus detection system (Thermo Fisher Scientific Shanghai Instrument Co., Ltd.) (Shanghai, China). The cycling conditions involved 40 cycles at 95 °C for 30 s, 95 °C for 5 s, and 60 °C for 34 s, followed by a melting curve phase. The cycle threshold (Ct) was set at a level where the exponential growth of PCR amplification among all the samples was approximately parallel. The reaction efficiency was calculated using the 2^−ΔΔCT^ method. Utilizing GAPDH as a reference, the target genes and Ct values were compared and the relative amounts in each sample were calculated (n = 6). Consequently, the relative gene expression levels of VEGF, PTEN, and Cyclin D1 were obtained, and the gene sequences are detailed in Table 1.

### 2.11. Immunohistochemical Assay (ICH)

The rat uterine tissues were fixed with 4% paraformaldehyde, dehydrated, paraffin-embedded, and cut into 5 μm sections. Following tissue section dewaxing and rehydration, the sections underwent antigen retrieval by immersion in, pH 9.0, Tris–EDTA buffer and were then exposed to microwave radiation at 100 °C for 10 minutes. Endogenous peroxidase was blocked using 3% hydrogen peroxide and incubated for 10 minutes in the dark to prevent nonspecific staining. The sections were incubated with rabbit monoclonal antibodies against PTEN (Bioss bs-0748R, 1:100 dilution), Cyclin D1 (Abcam, 1:200 dilution), and VEGF (Proteintech 19003-1-AP, 1:100 dilution) at 4 °C, overnight. After washing with PBS, the secondary antibody, goat anti-rabbit (1:200), was applied and incubated at 37 °C. Finally, the sections were visualized under a microscope, involving color development with DAB, rebluing, and dehydration. The analysis of the protein expression in each group was conducted using ImageJ 9.0 software and the relative protein expression was observed based on the mean optical density (MOD) value.

### 2.12. Data Analysis

Statistical analyses were conducted using IBM SPSS Statistics 26, GraphPad Prism (version 9.0), and Origin 64. The data are presented as the mean ± standard error (SEM). To compare the means across multiple groups, a one-way analysis of variance (ANOVA) was employed. Statistical significance was denoted by asterisks, with the significance levels indicated as follows: * *p* < 0.05, ** *p* < 0.01, *** *p* < 0.001, and **** *p* < 0.0001, respectively.

## 3. Results

### 3.1. Properties of the Creams

**Figure 1:**
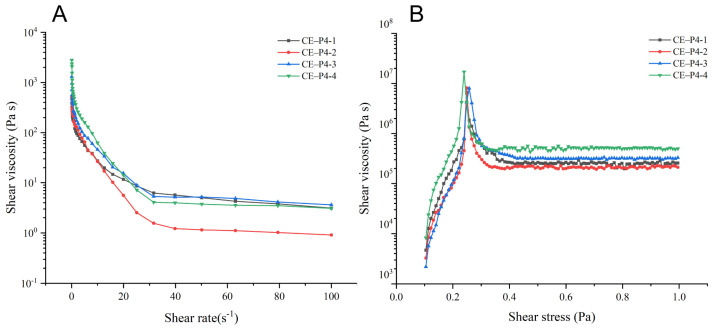
Cream rheological properties. (**A**): The flow curves of the four creams displayed typical features of shear-thinning, non-Newtonian fluids. (**B**): The yield stress of the four creams was found to be comparable.

The rheological properties of the four creams are illustrated in Figure 1. All the prepared creams exhibited a delicate appearance, with the drugs uniformly dispersed. The flow curves of the four creams displayed typical features of shear-thinning, non-Newtonian fluids (Figure 1A). Additionally, the yield stress of the four creams was found to be comparable (Figure 1B).

### 3.2. Vaginal Smear Cytology

**Figure 2:**
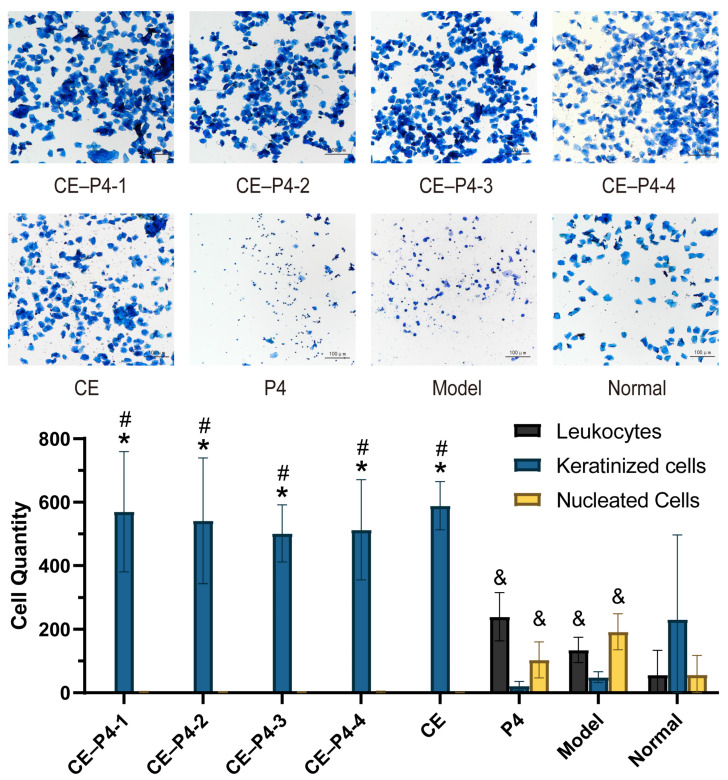
All four groups and the CE group had a large number of anucleated keratinized epithelial cells and were in estrus, while the P4 group and the model group showed a decrease in the number of cells in the vaginal smears, with a dramatic decline in keratinized cells and a predominance of leukocytes and nucleated epithelial cells, which were in the diestrus phase. The normal group had a complete estrus cycle, so there were no consistent patterns in terms of cell counts in the proestrus, estrus, metestrus, and diestrus phases, simultaneously. * *p* < 0.001 versus leukocytes; ^#^ *p* < 0.001 versus nucleated cells; ^&^ *p* < 0.001 versus keratinized cells using one-way ANOVA.

The results are depicted in Figure 2 and Table 2. Rats in the normal group exhibited a regular estrous cycle throughout the experiment, characterized by vaginal smears with cellular features of the four cycles being present. The existence of these cycles implies that the rats in the normal group were experiencing regular hormonal changes related to the estrous cycle and their reproductive system was operating regularly. Contrarily, the P4 group and the model group displayed a decrease in the total number of cells in the vaginal smears, with a predominance of leukocytes (a.k.a. neutrophils or polymorphonuclear cells) and nucleated epithelial cells, which were in the diestrus phase, and an abrupt reduction in keratinized epithelial cells. Due to the conjugated estrogen supply, the administration group and the CE group, notably, displayed oestrus characteristics, such as a high number of anucleated keratinized epithelial cells and an extremely low quantity of leukocytes and nucleated cells.

### 3.3. Uterine Coefficient, Serum Levels of Steroid Hormones

The rat uterine coefficient, calculated as the uterine weight (mg) divided by the body weight (g), indicates the uterus’s size relative to the body.

**Figure 3:**
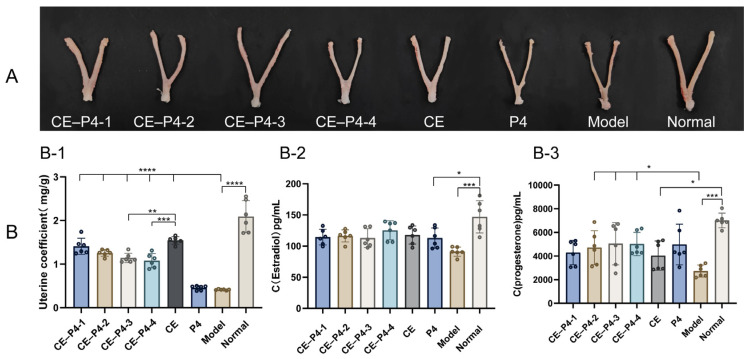
(**A**): Uterine morphology after administration. (**B**,**B-1**): uterine coefficient of rats; (**B-2**): serum estrogen content; (**B-3**): serum P4 content. (**B-1**): The uterine coefficients of the four co-administered dose groups showed a significant increase compared to the model group. The uterine coefficient was significantly decreased in the CE–P4-3 and CE–P4-4 groups compared to the CE group. (**B-2**): There were no significant differences in serum estradiol levels in the four dose groups compared to the CE group. (**B-3**): Blood P4 levels were significantly increased in the CE–P4-2, CE–P4-3, and CE–P4-4 groups compared to the model group, with no significant difference observed between the CE–P4-1 group and the model group. * *p* < 0.05, ** *p* < 0. 01, *** *p* < 0.001, and **** *p* < 0.0001.

The results in terms of the uterine coefficients are presented in Figure 3(B-1). The uterine coefficients of the P4 and model groups exhibited a significant decrease (*p* < 0.0001) compared to the normal group. In contrast, the uterine coefficients of the four co-administered dose groups showed a significant increase compared to the model group (*p* < 0.001). Furthermore, the uterine coefficient was significantly decreased in the CE–P4-3 and CE–P4-4 groups compared to the CE group (*p* < 0.001).

The results in terms of the serum steroid hormone levels are depicted in Figure 3(B-2,B-3). The estradiol concentrations were significantly lower in the P4 group (*p* < 0.01) and the model group (*p* < 0.001) compared to the normal group. However, there was no significant difference in the serum estradiol levels in the four co-administered dose groups compared to the model group (*p* > 0.05). Additionally, there were no significant differences in the serum estradiol levels in the four dose groups compared to the CE group (*p* > 0.05).

The concentration of P4 was significantly lower in the model group compared to the normal group (*p* < 0.001). However, blood P4 levels were significantly increased in the CE–P4-2, CE–P4-3, and CE–P4-4 groups compared to the model group (*p* < 0.05), with no significant difference observed between the CE–P4-1 group and the model group (*p* > 0.05).

### 3.4. Hematoxylin–Eosin Staining (HE)

**Figure 4:**
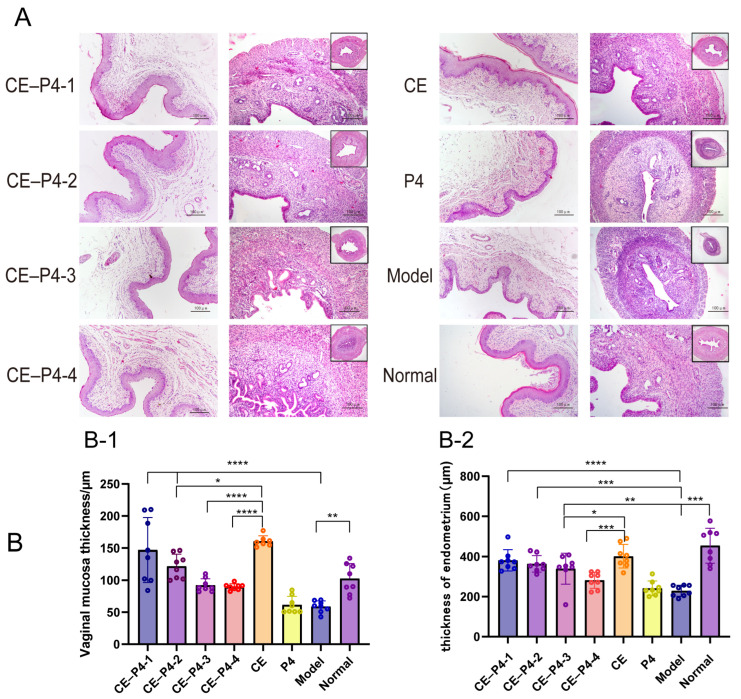
(**A**): Pathological slides of the uterus and vagina. (**B-1**): The thickness of the vaginal mucosa in the groups 1-4 decreased with the increase in the P4 dose. And the thickness of the vaginal mucosa was significantly decreased in the CE–P4-2, CE–P4-3, and CE–P4-4 groups compared to the CE group. (**B-2**): The endometrial thickness in the CE–P4-3 and CE–P4-4 groups was significantly decreased compared with the CE group (40×; 100×). * *p* < 0.05, ** *p* < 0. 01, *** *p* < 0.001, and **** *p* < 0.0001.

The results of the microscopic examinations after HE staining of the rat vaginal and uterine sections are presented in Figure 4A. In the normal group, the vaginal mucosa appeared intact, with mild keratinization and thickening. Conversely, the vagina of the model group did not exhibit keratinization and thickening, and inflammatory cell infiltration was evident. The vaginal mucosa of the CE group displayed noticeable keratinization and thickening; however, the overall thickness of the vaginal mucosa was lower than that of the normal group. Interestingly, the vaginal mucosa thickness decreased with increasing P4 doses in the CE–P4 groups.

As illustrated in Figure 4(B-1), compared to the model group, the CE group with co-administration of P4 exhibited a significant increase in the thickness of the vagina, an elevation in the maturation index of the vaginal epithelial cells, and a slight increase in secretion by the rats. However, the thickness of the vaginal mucosa was significantly decreased in the CE–P4-2, CE–P4-3, and CE–P4-4 groups compared to the CE group.

As illustrated in Figure 4(B-2), the results indicate a significant reduction in endometrial thickness in the model group compared to the normal group (*p* < 0.001). Conversely, the endometrial thickness showed a significant increase in the CE group compared to the model group (*p* < 0.001). Notably, the endometrial thickness in the CE–P4-3 and CE–P4-4 groups was significantly decreased (*p* < 0.05) compared with the CE group, with no significant differences observed compared to the normal group. There was a decreasing trend in the endometrial thickness with the increase in P4 dose. A specific morphological description of the endometrium in each group of rats is provided in Table 3.

**Figure 5:**
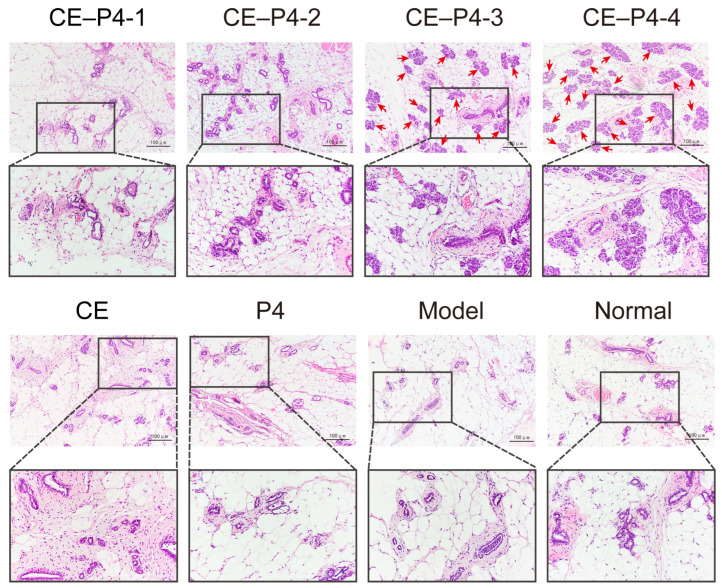
Compared with the normal group, the CE–P4-3 and CE–P4-4 groups showed significant hyperplasia of the mammary follicles and ductal epithelium, with a notable increase in the diameter and number of mammary follicles and lobules. None of the remaining groups exhibited hyperplasia compared to the normal group. Arrows show hyperplastic lobules and follicles (100×; 200×).

The results of the HE-staining, microscopic examinations of the rat mammary glands are depicted in Figure 5. In the normal group, the mammary tissue was in a quiescent state, with neatly arranged mammary ductal epithelial cells. The lumens of the ducts and alveoli were not dilated, and only a small number of mammary lobular follicles were present, with no mesenchymal hyperplasia.

Compared with the normal group, the CE–P4-3 and CE–P4-4 groups showed significant hyperplasia of the mammary follicles and ductal epithelium, with a notable increase in the diameter and number of mammary follicles and lobules. None of the remaining groups exhibited hyperplasia compared to the normal group.

### 3.5. Network Pharmacology

**Figure 6:**
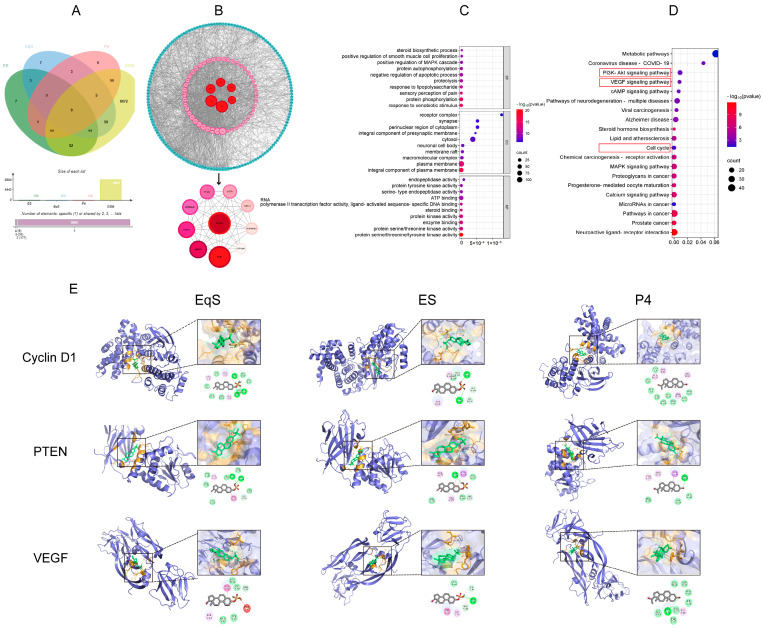
(**A**): Venn diagram; (**B**): PPI network interactions; (**C**): GO enrichment; (**D**): KEGG diagram; (**E**): molecular docking of ES, EqS, and P4 with Cyclin D1, PTEN, and VEGF, respectively.

Information for 100 genes related to the ES, EqS, and P4 drug targets was obtained from the SwissTargetPrediction and GeneCards databases. Additionally, information for 8884 genes related to the disease targets was gathered from the OMIM and DisGeNET databases. The Venn diagrams illustrating the overlap of the targets are shown in Figure 6A. A network was constructed using the Cytoscape 3.8.0 software, visualizing the “active ingredient–target” interactions, as presented in Figure 6B. The results demonstrate that ES, EqS, and P4 collectively interact with 108 targets through the network, exerting their effects on GSM. This network provides insights into the synergistic interactions between these active ingredients and their associated targets in the context of GSM. The results of the GO enrichment (*p* < 0.01) are illustrated in Figure 6C. There were 521 entries related to the biological processes, primarily associated with the phosphorylation of MAPK level proteins, the positive regulation of cell proliferation, and steroid synthesis, etc. Additionally, 90 entries were related to the cellular components, including receptor complexes, cell membranes, and plasma membrane components, etc. Furthermore, 166 entries were related to molecular functions, mainly associated with the activity of protein serine/threonine/kinases, steroids, protein tyrosine kinase activity, and other macromolecules. The KEGG enrichment results (*p* < 0.05) are presented in Figure 6D, indicating that the intersected targets were involved in signalling pathways, including, but not limited to, 127 KEGG signalling pathways. Based on the core targets screened by PPI and a review of the literature, the top 10 signalling pathways enriched by KEGG were identified, and the next pharmacodynamic validation will be carried out by selecting the PI3K–AKT signalling pathway, the VEGF signalling pathway, and the cell cycle signalling pathway. Three targets, Cyclin D1, PTEN, and VEGF, will be selected from the predicted pathways for further pharmacodynamic validation.

The obtained binding energies from molecular docking were consistently less than 6.0 kcal/mol, indicating a strong affinity between the core active components of CE and P4 with the key targets associated with therapeutic effects on GSM (Figure 6E). This robust affinity suggests the reliability of the network analysis results and supports the potential effectiveness of CE and P4 in treating GSM.

### 3.6. RT-qPCR

**Figure 7:**
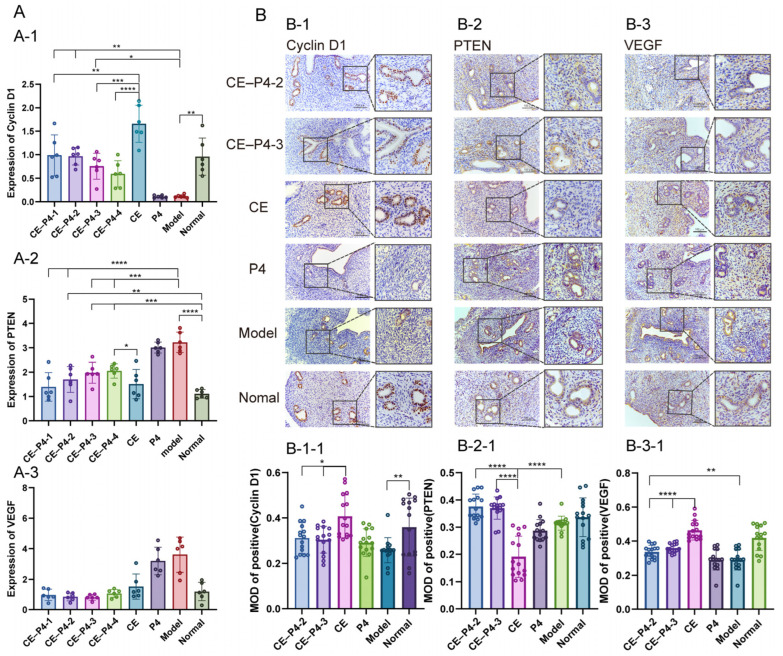
Expressions of Cyclin D1 in glandular epithelial cells, PTEN in stromal cells, and VEGF in stromal cells of endometrium, with IHC staining and RT-qPCR. (**A**): The expression levels of Cyclin D1, PTEN, and VEGF genes, respectively. (**A-1**): the relative expression of Cyclin D1 in the CE–P4-1, CE–P4-3, CE-P4–4 groups was significantly decreased compared with the CE group. (**A-2**): The relative expression of PTEN was significantly increased in the CE–P4-4 group compared with the CE group. (**A-3**): Compared with the CE group, the relative expression of VEGF in the CE–P4-1, CE–P4-2, CE–P4-3, and CE–P4-4 groups decreased, but the difference was not statistically significant. (**B-1**,**B-1-1**): The expression of Cyclin D1 in the CE–P4-2 and CE–P4-3 groups was significantly lower compared with CE group. (**B-2**,**B-2-1**): The expression of PTEN in the CE–P4-2 and CE–P4-3 groups was significantly higher compared with CE group. (**B-3**,**B-3-1**): The expression of VEGF in the CE–P4-2 and CE–P4-3 groups was significantly lower when compared with the CE group (100×; 200×). * *p* < 0.05, ** *p* < 0. 01, *** *p* < 0.001, and **** *p* < 0.0001.

The gene expression results are summarized as follows:

The Cyclin D1 gene expression is shown in Figure 7(A-1). The relative expression of Cyclin D1 in the model group was significantly lower compared to the normal group (*p* < 0.01). The relative expression of Cyclin D1 in the CE–P4-1, CE–P4-2, and CE–P4-3 groups was significantly higher compared to the model group (*p* < 0.01). Compared with the CE group, the relative expression of Cyclin D1 in the CE–P4-1, CE-P4-3, and CE–P4-4 groups was significantly decreased (*p* < 0.05).

The PTEN gene expression is shown in Figure 7(A-2). Compared with the normal group, the relative expression of PTEN in the model group was significantly increased (*p* < 0.001). The relative expression of PTEN was significantly increased in the CE–P4-4 group compared with the CE group (*p* < 0.05), while the CE–P4-1 group did not show an increase in PTEN expression.

The expression of the VEGF gene is shown in Figure 7(A-3). Compared with the CE group, the relative expression of VEGF in the CE–P4-1, CE–P4-2, CE–P4-3, and CE–P4-4 groups decreased, but the difference was not statistically significant (*p* > 0.05).

### 3.7. Immunohistochemical Staining (ICH)

The immunohistochemical staining results and mean optical density values for Cyclin D1, PTEN, and VEGF are summarized and depicted in Figure 7.

The Cyclin D1 immunohistochemical staining results are shown in Figure 7(B-1,B-1-1). The Cyclin D1 protein expression was significantly higher in the CE group compared to the model group (*p* < 0.01), and the CE–P4-2 and CE–P4-3 groups showed significantly lower Cyclin D1 expression compared to the CE group (*p* < 0.05).

The PTEN immunohistochemical staining results are shown in Figure 7(B-2,B-2-1). The PTEN protein expression was significantly lower in the CE group compared with the normal group. The model group, the CE–P4-2 group, and the CE–P4-3 group exhibited significantly higher PTEN expression than the CE group (*p* < 0.0001).

The results of the VEGF immunohistochemical staining results are shown in Figure 7(B-3,B-3-1). The expression of the VEGF protein was significantly increased in the CE group compared to the model group (*p* < 0.0001). The CE–P4-2 and CE–P4-3 groups showed significantly decreased VEGF protein expression compared to the CE group (*p* < 0.0001).

## 4. Discussion

The study results indicate that the vaginal administration of both CE monotherapy and the combination of CE with P4 effectively addresses GSM symptoms. The observed improvements include the restoration of vaginal cytology, the advancement of surface cells, the thickening of the vaginal mucosa, enhanced vaginal blood flow, fluid secretion, and an overall alleviation of GSM symptoms (see Figure 2 and Figure 4A,(B-1)). However, it is noteworthy that the administration of CE led to a significant increase in endometrial thickness compared to the model group (Figure 4(B-2)), indicating a potential risk of endometrial hyperplasia with prolonged use. This finding is consistent with the known risk of endometrial thickening associated with estrogen therapy. The presence of slight serum estradiol in the CE group (Figure 3(B-2)) further supports the possibility of systemic absorption and its impact on the endometrium. In contrast, the combination of CE with P4 significantly reduced endometrial thickness compared to the CE group (Figure 4(B-2)). This reduction in endometrial thickness may be attributed to the “uterine first-pass effect” of P4. Vaginal administration of P4 in the cream allows for rapid absorption and diffusion to the cervix and uterus, providing direct protection to the endometrium [18]. The dose-dependent increase in serum P4 levels after co-administration suggests effective absorption, with saturation observed in the CE–P4-3 and CE–P4-4 groups (Figure 3(B-3)). In conclusion, the results suggest that CE combined with P4 in a vaginal cream formulation can be an effective treatment for GSM, while mitigating the risk of endometrial hyperplasia associated with estrogen monotherapy.

To further investigate the mechanism of action of CE and P4 on the endometrium, this study first employed network pharmacology to identify potential signalling pathways (PI3K–AKT, VEGF, and cell cycle signalling pathways) related to CE, P4, and menopausal syndrome. Then, RT-qPCR and immunohistochemistry were used to partially validate some of the relevant target genes (Cyclin D1, PTEN, VEGF) associated with three signalling pathways. Finally, it analyzed the complex interactions between estrogen and P4 signalling pathways in the endometrium. Cyclin D1 plays a crucial role in cell cycle regulation, primarily promoting cell proliferation [19]. PTEN, an oncogenic factor and tumor suppressor gene, acts as an endogenous negative regulator of the Phosphatidylinositol 3-kinases (PI3K) signalling pathway [20,21]. The PI3K pathway regulates essential cellular processes, such as growth, apoptosis, and proliferation. The VEGF (Vascular Endothelial Growth Factor) signalling pathway involves the stimulation of new blood vessel formation. VEGF, also known as the Vascular Permeability Factor (VPF), is a glycoprotein that, upon binding to its receptor (VEGFR) on the cell surface, activates intracellular tyrosine kinases. This activation triggers a series of signalling cascade events related to angiogenesis and vasculogenesis [22]. By analyzing these target genes, this study aims to provide insights into the molecular mechanisms underlying the therapeutic effects of CE and P4 on the endometrium. This comprehensive approach, combining network pharmacology predictions with experimental validation, enhances the understanding of the potential pathways involved in the observed physiological responses.

Endometrial hyperplasia (EH) is characterized by abnormal proliferation of epithelial cells and glands in the endometrium [23]. It involves changes such as mild oedema and dilation of glandular lumens. As the endometrium thickens, it increases the risk of endometrial hyperplasia, which may be a precursor to endometrial cancer. In addition to the thickness of the endometrium, markers associated with endometrial endothelial proliferation may indicate the degree of endometrial hyperplasia [24]. Estrogen and P4 are among the signalling molecules that influence endometrial cell proliferation and/or apoptosis. Oestrogen can downregulate PTEN activity by increasing its phosphorylation [25]. This process activates the PI3K/AKT pathway and subsequently triggers the VEGF signalling pathway [26]. The VEGF signalling pathway can in turn initiate the PI3K/AKT pathway via Src signalling and increase the expression of Cyclin D1. This contributes to endometrial thickening and an increased uterine coefficient. Conversely, P4 inhibits the estrogen-induced PI3K/AKT signalling pathway by decreasing the phosphorylation of PTEN [25]. P4 also decreases the expression of VEGF and Cyclin D1, inhibiting angiogenesis in the myometrium and blocking the proliferation of uterine epithelial cells [27,28,29,30]. P4 has been found to effectively antagonize estrogen-induced endometrial proliferation by decreasing estrogen receptor expression, inducing cell differentiation and apoptosis [31]. The co-administration of P4 with estrogen increases the gene and protein expression of PTEN, while decreasing the gene and protein expression of Cyclin D1 and VEGF compared to estrogen monotherapy. This suggests that P4, when combined with estrogen, has the potential to reduce endometrial thickening and improve the prevention of endometrial cancer. Overall, the study underscores the intricate balance and interplay between estrogen and P4 signalling pathways in the endometrium, highlighting their implications for endometrial health and cancer prevention.

The study findings indicate that the vaginal administration of a high dose of P4, specifically in the CE–P4-3 and CE–P4-4 groups, led to marked hyperplasia in mammary gland follicles and mammary ductal epithelium. The increase in the diameter and number of mammary follicles and lobules suggest that P4 at higher doses may cause mammary gland hyperplasia. However, the medium (CE–P4-2) and low (CE–P4-1) doses of P4 appear to be relatively safe for the mammary glands. Notably, the CE–P4-1 group did not significantly reduce endometrial thickness compared to the CE group. The overall implication is that the co-administration of the second dose group (CE–P4-2) could potentially mitigate endometrial side effects caused by long-term CE use, while maintaining safety in terms of the mammary glands. It is important to acknowledge that while the study focused on the effects of CE and P4 on the endometrium, the impact and underlying mechanisms of these hormones on the mammary glands were not explored comprehensively. Future research will further delve into the effects and mechanisms of CE and P4 on the mammary glands in order to provide a more comprehensive understanding.

## 5. Conclusions

In summary, the study results indicate that the vaginal administration of CE combined with P4 for 12 days led to a significant reduction in the expression of gene proteins related to the uterine coefficient, Cyclin D1, and VEGF, in the four dosage groups (CE–P4-1, CE–P4-2, CE–P4-3, and CE–P4-4) compared to the group receiving the CE monotherapy. Conversely, there was a significant increase in the expression of gene proteins related to PTEN in the combined dosage groups, suggesting that the addition of P4 can effectively inhibit endometrial hyperplasia, while treating genitourinary syndrome of menopause (GSM) and improve the potential to prevent endometrial cancer.

The findings suggest that the compound vaginal preparation containing P4 has a protective effect on the endometrium, while maintaining the therapeutic efficacy of CE in the vagina. This protective effect has been preliminarily supported at the signalling pathway level. Notably, the CE–P4-2 group, which includes a cream containing CE (0.625 mg/g) and P4 (80 mg/g), showed promise in reducing the endometrial side effects associated with long-term CE use, while ensuring the safety of the mammary glands. This specific dosage combination could serve as a foundational specification for the subsequent development of a CE–P4 compound vaginal cream.

## Figures and Tables

**Table 1:** Primer sequence.

Gene Symbol	Forward Primer	Reverse Primer
GAPDH	CAGGGCTGCCTTCTCTTGTG	TGCACCCACGACAGAAGG
VEGF	TGCACCCACGACAGAAGG	GCACACAGGACGGCTTGA
Cyclin D1	CAAGTGTGACCCGGACTGC	GACCAGCTTCTTCCTCCACTT
PTEN	CAATGTTCAGTGGCGGAACTT	GGCAATGGCTGAGGGAACT

**Table 2:** Comparison of cell populations throughout the estrous cycle of the groups (x ± SD, n = 6).

Group	Leukocytes	Keratinized Cells	Nucleated Cells
CE–P4-1	1.30 ± 1.64	570 ± 109.62 *^#^	2.91 ± 3.14
CE–P4-2	1.5 ± 0.71	541.56 ± 198.17 *^#^	3.00 ± 1.94
CE–P4-3	1.33 ± 0.58	501.78 ± 90.11 *^#^	2.80 ± 3.03
CE–P4-4	3.67 ± 3.39	513.000 ± 157.90 *^#^	5.50 ± 4.48
CE	5.00 ± 1.00	673.50 ± 205.49 *^#^	3.00 ± 2.83
P4	239.67 ± 75.81 ^&^	21.61 ± 14.12	103.44 ± 56.65 ^&^
Model	192.33 ± 56.59 ^&^	16.50 ± 9.66	130.11 ± 50.14 ^&^
Normal	58.88 ± 78.79	230.78 ± 266.10	57.06 ± 60.44

* *p* < 0.001 versus leukocytes; ^#^ *p* < 0.001 versus nucleated cells; ^&^*p* < 0.001 versus keratinized cells using one-way ANOVA.

**Table 3:** Description of the endometrial morphology in each group.

Clusters	Organizational patterns	Cellular morphology
CE–P4-1 CE–P4-2 CE–P4-3 CE–P4-4	The endometrium was essentially intact, with significant thinning of the endometrium and a reduced number of glands compared to the normal group.	The morphology of the endothelial epithelial cells tended to be small and cylindrical as the P4 increased.
CE	Uterine horns were observed by the naked eye to be dilated due to retention of the secretion.	The endothelial epithelial cells were miniaturized, the endothelial lamina propria and the muscularis propria showed pheochromocytosis, and the endothelial lamina propria was accompanied by mild oedematous changes. The glandular lumen was mildly dilated. The glandular epithelium was cylindrical or hypercolumnar, with increased basophilia and enlarged nuclei.
P4 model	The maturation of the uterus was inhibited and miniaturized. Histological observation: the uterus was reduced in size.	The endometrial epithelial cells and uterine glandular epithelial cells were small and cylindrical, with a nuclear/plasmic ratio of 1.5 or less, vacuolization, necrosis, and nuclear schizophrenia were not evident. The endometrial mesenchymal cells were spindle-shaped, with less cytoplasm, and the smooth muscle cells of the myometrium were smaller.
Normal	The endometrium was intact with a normal number of glands; dilatation of the uterine lumen was mostly observed as the animal’s uterus varied with the normal sexual cycle, especially during the pre-oestrus period.	The cell morphology was normal.

## Data Availability

The data are contained within the article.
